# Two cortical representations of voice control are differentially involved in speech fluency

**DOI:** 10.1093/braincomms/fcaa232

**Published:** 2021-01-05

**Authors:** Nicole E Neef, Annika Primaßin, Alexander Wolff von Gudenberg, Peter Dechent, Christian Riedel, Walter Paulus, Martin Sommer

**Affiliations:** 1 Department of Clinical Neurophysiology, Georg August University, Göttingen 37075, Germany; 2 Department of Diagnostic and Interventional Neuroradiology, Georg August University, Göttingen 37075, Germany; 3 Institut der Kasseler Stottertherapie, Bad Emstal, Germany; 4 Department of Cognitive Neurology, MR Research in Neurosciences, Georg August University, Göttingen 37075, Germany; 5 Department of Neurology, Georg August University, Göttingen 37075, Germany

**Keywords:** speech, stuttering, voice control, diffusion imaging, white-matter pathways

## Abstract

Recent studies have identified two distinct cortical representations of voice control in humans, the ventral and the dorsal laryngeal motor cortex. Strikingly, while persistent developmental stuttering has been linked to a white-matter deficit in the ventral laryngeal motor cortex, intensive fluency-shaping intervention modulated the functional connectivity of the dorsal laryngeal motor cortical network. Currently, it is unknown whether the underlying structural network organization of these two laryngeal representations is distinct or differently shaped by stuttering intervention. Using probabilistic diffusion tractography in 22 individuals who stutter and participated in a fluency shaping intervention, in 18 individuals who stutter and did not participate in the intervention and in 28 control participants, we here compare structural networks of the dorsal laryngeal motor cortex and the ventral laryngeal motor cortex and test intervention-related white-matter changes. We show (i) that all participants have weaker ventral laryngeal motor cortex connections compared to the dorsal laryngeal motor cortex network, regardless of speech fluency, (ii) connections of the ventral laryngeal motor cortex were stronger in fluent speakers, (iii) the connectivity profile of the ventral laryngeal motor cortex predicted stuttering severity (iv) but the ventral laryngeal motor cortex network is resistant to a fluency shaping intervention. Our findings substantiate a weaker structural organization of the ventral laryngeal motor cortical network in developmental stuttering and imply that assisted recovery supports neural compensation rather than normalization. Moreover, the resulting dissociation provides evidence for functionally segregated roles of the ventral laryngeal motor cortical and dorsal laryngeal motor cortical networks.

## Graphical Abstract

**Graphical Abstract:**
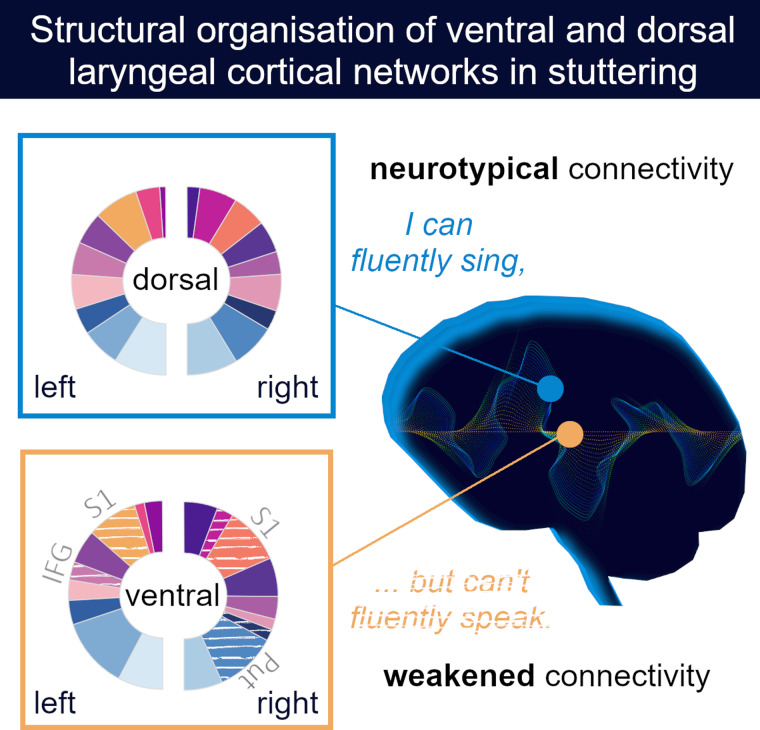

## Teaser

Humans are most advanced in producing song and speech, and the dorsal and ventral laryngeal representation are hypothesized to constitute specialized parts of the speech control circuits. Here, Neef *et al.* find that the dorsal area has an overall stronger structural network and that only the ventral network is weakened in persistent stuttering.

## Introduction

The human precentral gyrus comprises two representations of voice control. The dorsal laryngeal motor cortex (dLMC) is located between the cortical representations of the lips and the hands (dLMC) (Rödel *et al.*, 2004; Brown *et al.*, 2008; Olthoff *et al.*, 2008; Bouchard *et al.*, 2013; Belyk and Brown, 2017). The ventral laryngeal motor cortex (vLMC) occupies parts of the subcentral gyrus and the rolandic operculum (Foerster, 1931; Bouchard *et al.*, 2013; Breshears *et al.*, 2015). Partly due to technical challenges with investigating speech and singing *in vivo* in the human brain, only little is known about the structural and functional organization of dLMC and vLMC networks (Simonyan, 2014; Kumar *et al.*, 2016; Belyk and Brown, 2017).

Only recently, high-density cortical recordings from the two laryngeal representations segregated two distinct functions of the larynx, the encoding of voicing and pitch. Neural populations in both regions encode articulatory voicing and contribute to the differentiation of voiced and voiceless speech sounds. However, vocal pitch in human speech and singing seems to be selectively encoded and controlled via dLMC neurons (Dichter *et al.*, 2018). Specifically, Dichter *et al.* (2018) observed that the direct focal electrical stimulation of the dLMC evoked laryngeal movements and involuntary vocalization, making a causal role of dLMC in the feedforward control of vocal pitch most likely, whereas there were no instances where stimulating the vLMC elicited vocalization. Other recent studies have shown speech arrest (Chang *et al.*, 2017) or larynx paralysis (Roux *et al*., 2020) from stimulating in the vLMC location. In their seminal work from the 1950s, Penfield and Roberts (1959) already documented vocalizations during the stimulation of the precentral and postcentral gyri for lips, jaw and tongue. Notably, in Penfield’s account, the term vocalization did not differentiate between sustained and interrupted vowel cries and the larynx representation has not been assigned to a specific location in the precentral gyrus.

Electrical (Foerster, 1931; Penfield and Roberts, 1959; Breshears *et al.*, 2015; Chang *et al.*, 2017; Dichter *et al.*, 2018; Roux *et al*., 2020) and transcranial stimulation studies (Rödel *et al.*, 2004; Chen *et al.*, 2017) deliver valuable insights into the functional role of anatomical patches of the cortex. Still, its exploration is restricted to surface areas and cytoarchitectonic classification is uncertain. Deeper structures such as the sulcal regions of the precentral gyrus are better accessible via magnetic resonance imaging (MRI) techniques. A recent ultra-high field functional MRI study tested the involvement of dLMC and vLMC neurons in respiratory motor control by having participants whistle and sing simple melodies (Belyk *et al*., 2020). Both LMC regions were involved during singing as well as whistling, suggesting their functional contribution to voicing and expiration. A different study from the same group tested the somatotopic representation of larynx and jaw muscles, demonstrating again a proximity in both precentral sites (Brown *et al*., 2020), but no functional segregation.

Previous *in vivo* imaging studies of the structural connectivity of the laryngeal motor cortex in humans were restricted to the dLMC (Simonyan *et al.*, 2009; Kumar *et al.*, 2016). Probabilistic diffusion tractography showed that the human left and right dLMC have moderate connections with the inferior frontal gyrus, superior temporal gyrus, supplementary motor area (SMA), caudate nucleus, putamen and globus pallidus, and particularly dense projections with the somatosensory cortex and the inferior parietal cortex (Kumar *et al.*, 2016). All these connections are anatomically plausible and validated by neuroanatomical tract-tracing studies of the laryngeal motor cortex representation in the rhesus monkey (Simonyan and Jürgens, 2002, 2003, 2005a,b). However, compared to macaque, the human dLMC network showed stronger connections with brain regions involved in the processing of sensory information and feedback, i.e. the primary somatosensory cortex, inferior parietal lobe and superior temporal gyrus (Kumar *et al.*, 2016). The authors discuss this finding with the idea that in particular the enhanced connectivity of the dLMC with parietotemporal regions that are involved in sensorimotor integration might have contributed to the development of the sophisticated vocal motor control that is essential for fluent speech production. The dLMC is part of the vast vocal tract sensorimotor cortex and fluent speech production involves the whole orofacial homunculus and in particular, the sub-central gyrus and the rolandic operculum. This ventral extension of the central sulcus harbours the vLMC (Foerster, 1936; Bouchard *et al.*, 2013; Breshears *et al.*, 2015). Only recently, studies have started targeting and differentiating findings from the dorsal and the ventral motor representation of voice control (Dichter *et al.*, 2018; Belyk *et al*., 2020). Ultimately, fundamental questions exist about what is the structural organization of the vLMC network, does it differ from dLMC network organization and will the learning of a changed voicing behavior reorganize the structural network formation within both networks?

One intriguing approach to scrutinize structural network characteristics of the dual cortical laryngeal motor representations is the study of network organization in persistent developmental stuttering. Persistent developmental stuttering is a speech fluency disorder with a complex genetic basis (Kraft and Yairi, 2012). Most often, it occurs in early childhood without obvious reason and persists in about 1% of the adults preferably in the male population (Yairi and Ambrose, 2013). Stuttering is evident in sound and syllable repetitions, sound prolongations and speech blocks, which demonstrates the difficulties of affected individuals to initiate, control and terminate speech movements (Guenther, 2016). These speech motor signs are often accompanied by physical concomitants such as facial grimacing, head and limb movements. Experience of stuttering can cause avoidance behaviors and social anxieties and may impact social well-being, professional career and socio-economic status (Craig and Tran, 2014). It is widely assumed that stuttering results from a neurofunctional deficit of speech motor planning, sequencing and sensorimotor integration involving system-wide correlates of the speech function, in particular left perisylvian speech areas, basal ganglia and cerebellum (Ludlow and Loucks, 2003; Craig-McQuaide *et al.*, 2014; Neef *et al.*, 2015; Etchell *et al*., 2017; Chang *et al.*, 2018; Connally *et al.*, 2018; Chang and Guenther, 2019). Strikingly, one robust neural trait marker of persistent developmental stuttering is a white-matter deficit adjacent to the left vLMC (Sommer *et al.*, 2002; Watkins *et al.*, 2008; Chang *et al.*, 2010; Neef *et al.*, 2015). Involved fiber tracts connect fronto-parietal/temporal circuit that promotes speech production (Hickok and Poeppel, 2007; Friederici, 2011; Hickok, 2012). A disruption of these connections might disturb speech signal transmission and thus, hamper speech fluency (Sommer *et al.*, 2002). On the contrary to the white-matter deficit in the left vLMC, fluency shaping, a stuttering intervention that involves learning to speak with reduced pitch modulation and voicing complexity (Euler *et al.*, 2009), synchronizes task-free brain activity between the left dLMC and the sensorimotor brain regions (Korzeczek *et al*., 2020). Briefly summarized, persistent stuttering is linked to a white-matter deficit in the left vLMC, whereas assisted recovery from stuttering via fluency shaping is linked to an increased functional connectivity of the dLMC.

Currently, it is an open question, whether intensive learning of a new voicing pattern will shape the structural organization of the two laryngeal motor representations and if so, whether neuroplasticity is similar or different between these two networks. In addition, it is, in general, unclear, whether dLMC and vLMC have structural connectivity patterns that are distinct or comparable, independent from the speech fluency of studied individuals. Therefore, this study, we re-examined diffusion MRI data of adults who do not stutter (AWNS), adults who stutter and who participated in an 11-month intensive fluency-shaping intervention (AWS+), and adults who stutter but did not participate in the intervention (AWS−). We used probabilistic diffusion tracking with bilateral seeds in the dLMC and vLMC and quantified respective connection probabilities with the somatosensory cortex, inferior parietal cortex, inferior frontal gyrus, superior temporal gyrus, SMA, caudate nucleus, putamen and globus pallidus (Kumar *et al.*, 2016). Neuroanatomical tract-tracing studies of the laryngeal motor cortex representation in the rhesus monkey show, in addition, hard-wired reciprocal connections with the thalamus, anterior cingulate cortex and mid-cingulate cortex (Jürgens, 2002; Simonyan *et al.*, 2009; Price, 2012; Simonyan and Fuertinger, 2015). However, in a previous study, these target regions revealed no significant proportions of projections when applying probabilistic diffusion tracking (Kumar *et al.*, 2016) and thus were not included in our analyses. We used an exploratory statistical approach, i.e. mixed-model ANCOVA, to determine the influence of seed, hemisphere, target, time and group. Furthermore, we tested whether stuttering severity was predicted by the structural network profiles of dLMC and vLMC, respectively.

## Materials and methods

### Participants

Current data were derived from a dissertation project (Primaßin, 2019) that evaluated the long-term effects of an intensive stuttering intervention on white-matter integrity and task-related brain activity. Here, we analyzed diffusion MRI data sets of 22 adults with stuttering who took part in a fluency-shaping program (AWS+, two females, mean age, 25.6 ± 11.7 SD). The program, the Kasseler Stotter Therapie (Euler *et al.*, 2009), incorporates fluency shaping with computer-assisted biofeedback during a 2-week on-site and a 1-year follow-up treatment. Fluency shaping changes the patterns of vocalization, articulation and respiration, resulting in prolonged speech, soft voice onsets of initial phonemes and a smooth transition between sounds. Furthermore, we analyzed data of 18 adults with stuttering who did not participate in any intervention during this study (AWS−, two females, mean age, 34.8 ± 7.0 SD), and of 28 adults without stuttering (AWNS, four females, mean age, 25.1 ± 7.4 SD). Participants completed two MRI sessions 11.5 ± 1.1 SD month apart and received an allowance for their expenses. All were monolingual native speakers of German, reported normal (or-corrected-to normal) vision and no history of hearing, speech, language or neurological deficits apart from stuttering in the AWS groups, drug abuse or medications that act on the central nervous system. The groups were matched for sex and handedness (Oldfield, 1971). AWS− were older and had a higher education score than participants in the two other groups (Table 1). Education and age were correlated with *r *=* *0.483, *P *<* *0.001, and therefore only age was considered as a covariate in all statistical analyses.

**Table 1:** Demographic information of participants

	Intervention group	Stuttering controls	Fluent controls	Test-statistics (*df*)	Two-sided *P*-value
*n*	22	18	28
Age (years)	25.6 ± 11.7	34.8 ± 7.0^*^	25.1 ± 7.4	7.58 (2, 65)^a^	0.001
Sex ratio	20:2	16:2	24:4	−^b^	0.89
Education^c^	2 (1.0)^#^	6 (3.0)	3 (2.8)	27.49 (12)^d^	<0.001
Handedness	91 (12)	91 (33)	100 (33)	0.04 (2,68)^d^	0.98
SSI-4 at T1	25 (14.3)	14 (11.3)	−	2.56^e^	0.010
SSI-4 at T2	9 (10.5)	12.5 (11.0)	−	−1.31^e^	0.194
OASES at T1	3.0 (0.6)	2.0 (0.4)	−	4.70^e^	<0.001
OASES at T2	1.9 (0.5)	2.0 (0.5)	−	−0.65^e^	0.516
Onset (years)	4.8 ± 3.0	5.0 ± 3.6	−	0.22^e^	0.839
Interval (months)	11.6 ± 1.0	11.6 ± 1.4	11.4 ± 0.8	0.95 (2)^d^	0.623

Interval/ratio-scaled variables are presented as mean ± standard deviation. Ordinal-scaled variables are presented as median (interquartile range).

* Significantly different from both other groups in *post-hoc* comparisons (*P* < 0.001).

# Significantly different from stuttering controls (*P* < 0.001)

a One-way independent ANOVA.

b Fisher’s exact test.

c 1 = still attending school, 2 = school, 3 = high school, 4 = <2 years college, 5 = 2 years of college, 6 = 4 years of college, 7 = post-graduate.

d Kruskal–Wallis test.

e Mann–Whitney test.

The ethical review board of the University Medical Center Göttingen, Georg August University Göttingen, Germany, approved the study, and all participants provided written informed consent, according to the Declaration of Helsinki, before participation.

Speech fluency of all participants, determined by using the Stuttering Severity Index (SSI-4, (Riley *et al.*, 2004), was assessed prior to each MRI session. As part of this assessment, each AWS was video recorded while reading aloud and speaking with an experimenter. Two certified speech-language pathologist (one of them was A.P.) then rated the frequency and durations of the stuttered syllables and the presence of physical concomitants. The raters were not blinded to group assignment and time. At test time point one (T1), stuttering severity in the AWS+ group ranged from 7 to 39, with a median of 25 and an interquartile range of 15–31. Five of the 22 AWS+ were categorized as very mild, five as mild, six as moderate, three as severe, two as very severe and one with an SSI-4 total score of 7 was not classified. The stuttering severity in the AWS− group ranged from 4 to 42, with a median of 14 and an interquartile range of 7–21. Eight of the 18 AWS− were categorized as very mild, two as mild, one as moderate, one as severe, one as very severe and five with SSI-4 scores between four and seven were not classified. Fluency shaping reduced stuttering severity in AWS+, (Primaßin, 2019; Korzeczek *et al*., 2020). Accordingly, at test time point two (T2), stuttering severity in the AWS+ group ranged from 1 to 37, with a median of 9 and an interquartile range of 5–16. After therapy, five of the AWS+ group were categorized as very mild, two as mild, one as moderate, one as very severe and 13 with an SSI-4 score of <9 were not classified. Before intervention, stuttering severity was more severe in AWS+ as compared to AWS−. Similarly, the self-assessment of the psycho-social impact of stuttering (Overall Assessment of the Speaker’s Experience of Stuttering, OASES) (Yaruss and Quesal, 2014) indicated that the participants of the intervention group suffered more from stuttering than stuttering controls. These group differences vanished after stuttering intervention (Table 1).

### Image acquisition

MRI data were acquired in a 3-Tesla Siemens Magnetom Tim Trio scanner (Erlangen, Germany) using an eight-channel phased-array head coil at the University Medical Center Göttingen, Germany. Sagittal T1-weighted structural data were acquired with a 3D turbo fast low-angle shot (FLASH) sequence (TR = 2250 ms, TE = 3.26 ms, TI = 900 ms, flip angle = 9°, 256 mm FoV and 7/8 Fourier phase encoding) as whole-brain anatomical reference data at a spatial resolution of 1 × 1 × 1 mm³ voxel size (256 × 256 matrix). Diffusion-weighted MRI data were acquired with a spin-echo EPI sequence (TR = 10 100 ms, TE = 93 ms, parallel acquisition factor 2, 6/8 Fourier phase encoding, 243 mm FoV, acquisition matrix: 128 × 128, 74 slices, voxel size 1.9 × 1.9 x 1.9 mm³) sampling 64 image volumes with diffusion weighting along 64 diffusion directions (*b* = 1000 s/mm^2^) and one reference image without diffusion weighting. Participants lay in supine position in the scanner and wore headphones for noise protection, and MR-compatible LCD goggles (VisuaStim XGA, Resonance Technology Inc., Northridge, CA, USA).

### MRI data analysis

**Figure 1:**
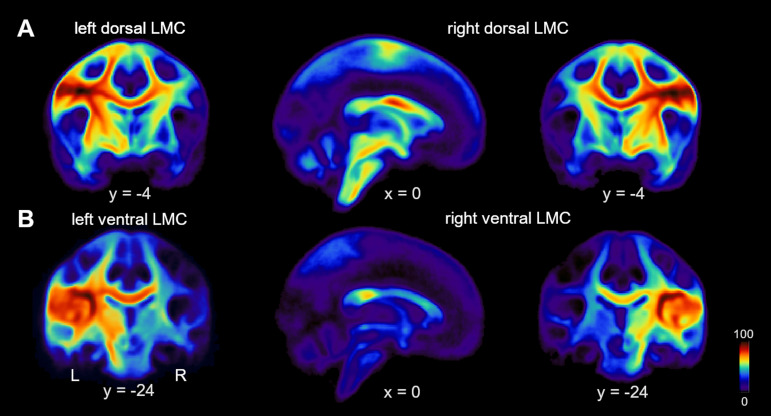
**Connection probability of two distinct larynx cortical representations.** (**A**) Population probability maps showing the likelihood of structural connectivity of the left and right dLMC and (**B**) left and right vLMC with dark-red marking 100% and dark-blue 0% connection probabilities.

Diffusion-weighted (d) MRI images were processed with FSL, https://www.fmrbi.ox.ac.uk/fsl/fslwiki (Jenkinson *et al.*, 2012; 31 December 2020, date last accessed). Images were corrected for eddy currents and head motion by using affine registration to the non-diffusion volumes. Probabilistic tractography was performed in the native dMRI space. We computed voxel-wise estimates of the fiber orientation distribution of up to two fiber orientations with the FSL function bedpost (Behrens *et al.*, 2007; Jbabdi *et al.*, 2012). Seed and target masks were 3-mm spheres. Coordinates of seeds of the dLMC were derived from a previous quantitative meta-analysis (Kumar *et al.*, 2016), but transformed via GingerALE (http://www.brainmap.org/ale/; 31 December 2020, date last accessed) from Talairach–Tournoux space to MNI space (*x* = −46, *y* = −12, *z* = 34; *x* = 48, *y* = −9, *z* = 36), and shifted from the grey matter in the anterior wall of the central sulcus (*x* = −45, *y* = −14, *z* = 33; *x* = 44, *y* = −12, *z* = 35) to the white matter in the pre-central gyrus (*x* = −47, *y* = −4, *z* = 34; *x* = 45, *y* = −3, *z* = 35) according to the FSL_HCP1065_FA _1mm standard image. Seeds of the vLMC were placed at (*x* = −46, *y* = −16, *z* = 19; *x* = 46, *y* = −16, *z* = 19) in the white matter adjacent to the subcentral sulcus and the rolandic operculum (Sommer *et al.*, 2002). Coordinates of the target masks of the SMA, posterior portion of the inferior frontal gyrus pars opercularis (pIFGop), anterior portion of the inferior frontal gyrus pars opercularis (aIFGop), primary somatosensory cortex (S1), inferior parietal lobe (IPL), putamen (Put), caudate nucleus (Caud) and globus pallidus (Gp) (Table 2) were derived from a previous study with probabilistic diffusion tractography of the LMC in humans (Kumar *et al.*, 2016). The original coordinate of the left SMA did not lay in the FSL_HCP1065_FA _1mm template. For this reason, we mirrored the original coordinate, given for the right SMA by multiplying x with -1. Considering the coordinates of the anterior superior temporal gyrus (aSTG), the original coordinates were associated with the left insular cortex (*x* = −41, *y* = −4, *z* = 0) and with the right inferior frontal gyrus (BA45) (*x* = 52, *y* = 11, *z* = 2) according to the Harvard–Oxford cortical structural atlas and the Jülich histological atlas (https://fsl.fmrib.ox.ac.uk/fsl/fslwiki/Atlases). For this reason, we located aSTG coordinates at the maximum of the respective tissue probability (*x* = −56, *y* = 0, *z* = −9) in the left aSTG and mirrored that coordinate by multiplying x with -1 −1 (*x* = 56, *y* = 0, *z* = −9). Seed and target coordinates were warped to the native FA map via the inverse warp field generated with FNIRT (Andersson *et al.*, 2010) and enlarged to 3-mm spheres. We used modified Euler streamlining, distance correction and 100 000 samples per voxel within the FSL function probtrackx2 with 36 pairs of seed and target mask. Target mask determined both waypoint and termination mask to compute the structural connectivity. All 36 analyses were calculated intra-hemispherically and separately for each pair of seed and target region. The ‘connectivity index’ was determined from the number of sample streamlines from each seed that reached the target. We normalized the connectivity index by dividing the logarithm of the number of streamlines from a given seed that reached the target (i.e. numeric output of the tractography algorithm given as waytotal) by the logarithm of the product of the number of generated sample streamlines in each seed voxel (100 000) and the number of voxels in the seed mask, *n* = 19. The logarithmic scaling transformed the connectivity index into a normally distributed variable with a range between 0 and 1. LMC population probability maps for probabilistic diffusion tracking without target masks are shown in Fig. 1.

**Table 2:** MNI coordinates of target regions

Region	Hemisphere	Tissue probability	MNI coordinate
S1	L	OP4 28%, 3b 24%, 3a 23%	[−56 −6 19]
	R	OP4 47%, OP3 10%, 3b 10%	[58 −4 16]
pIFGop	L	44 36%, 45 8%	[−51 9 12]
	R	44 37%, 45 10%, OP4 10%	[54 10 7]
aIFGop	L	44 54%, 45 20%	[−55 13 21]
	R	45 90%, 44 35 %4	[55 23 15]
IPL	L	PFm 27%, hIP1 19%, hIP3 10%	[−43 −54 29]
	R	Pga 18%, hIP1 16%	[44 −52 32]
aSTG	L	aSTG^a^ 71%	[−56 0 −9]
	R	aSTG^a^ 56%	[56 0 −9]
SMA	L	6 76%	[−7 1 70]
	R	6 100%	[7 1 70]
Put	L	Put^b^ 90%	[−31 −11 −1]
	R	Put^b^ 87%	[31 −13 5]
Caud	L	Caud^b^ 22%	[−20 17 9]
	R	Caud^b^ 35%	[7 6 0]
Gp	L	Pallidum^b^ 43%	[−24 −14 5]
	R	Pallidum^b^ 33%	[25 −14 5]

Tissue probabilities for the reported coordinates were given in percent and were derived from Jülich Histological Atlas.

a Harvard–Oxford Cortical Structural Atlas.

b Harvard–Oxford Subcortical Structural Atlas

*Abbreviations*: aIFGop, anterior inferior frontal gyrus pars opercularis; aSTG, anterior superior temporal gyrus; Caud, nucleus caudatus; Gp, globus pallidus; hIP, intra-parietal sulcus area; IPL, inferior parietal lobule; OP, parietal operculum; PFm, inferior parietal lobule area; Pga, inferior parietal lobule area; pIFGop, posterior inferior frontal gyrus pars opercularis; Put, putamen; S1, somatosensory cortex; SMA, supplementary motor area

### Statistical analyses

For quantitative between-group analysis of the human dLMC and vLMC network, we used the connectivity indices revealed for each seed-to-target pair within one mixed-model ANCOVA. We modelled Group (AWS+, AWS− and AMNS) as between-subjects factor, Time (T1, T2), Seed (dLMv and vLMV), Hemisphere (left hemisphere and right hemisphere) and Target region (SMA, pIFGop, aIFGop, S1, IPL, STG, Put, Caud and Gp) as repeated measures within-subjects factors and Age as a covariate. If the main effect of Seed was significant, the follow-up *post-hoc* ANCOVAs examined the two LMC networks separately with Group (AWS+, AWS− and AMNS) as between-subjects factor, Time (T1, T2), Hemisphere (left hemisphere and right hemisphere) and Target region (SMA, aIFGop, pIFGop, S1, IPL, STG, Put, Caud and Gp) as repeated measures within-subjects factors, and Age as a covariate.

We assessed tract lateralization by using the laterality index calculated as (connectivity index in the right hemisphere-connectivity index in the left hemisphere)/(connectivity index in the right hemisphere + connectivity index in the left hemisphere). A positive value indicates a larger connectivity index for the right compared to the left hemisphere, whereas a negative value indicates a smaller connectivity index for the right compared to the left hemisphere. We tested the significance of the laterality index by calculating two-sided one-sample *t*-tests against a mean value of zero and report significant lateralization at *P* < 0.05 (Bonferroni-corrected). Furthermore, we compared laterality indices between seeds with paired *t*-tests and report significant differences at *P* < 0.05 (Bonferroni-corrected).

Hierarchical regressions were used to predict pre-intervention speech fluency (SSI-4 total score) of affected individuals from structural connectivity profiles of dLMC and vLMC, respectively. The first step comprised demographic factors (age, sex and handedness) and the second step comprised connection probabilities; thus, the models estimate what percentage of variance in structural connectivity accounts for speech fluency above and beyond demographics.

### Data availability

The data that support the findings of this study are available from the corresponding author upon reasonable request.

## Results

### Across network analysis

The 3 × 2 × 2 × 2 × 9 ANCOVA performed on the connection strength of dLMC and vLMC revealed a significant main effect of seed, *F*(1,64) = 11.821, *P* = 0.001, $\eta_{p}^{2}$ = 0.156, such that dLMC, mean (*M*) = 0.377, 95% confidence interval (CI) [0.367–0.388] is characterized with an overall higher connectivity than vLMC, *M* = 0.315, 95% CI = [0.306–0.325]. There was also a trend for an interaction of Seed × Group, *F*(2,64) = 2.728, *P* = 0.0073, $\eta_{p}^{2}$ =0.079. In addition, there were a main effect of Target region, a main effect of Age and an interaction of Seed × Hemisphere, an interaction of Seed × Target region, an interaction of Hemisphere × Target region, an interaction of Hemisphere × Target region × Age, an interaction of Seed × Hemisphere × Target region and an interaction of Seed × Hemisphere × Target region × Age (all reported in Table 3). The four-way interaction indicates that the structural connectivity of dLMC and vLMC varies, depending on hemisphere and target region and that this variance is, in addition, modulated by age. There was no main effect of Time or interaction of Time × Seed × Group or of Time × Seed × Target region × Group, indicating no change of the structural connectivity of the two larynx areas over time and no intervention-induced neuroplasticity at this global analysis level.

**Table 3:** Results of the global mixed-model ANCOVA

	*df*	*F*	*P*-value	$\eta_{p}^{2}$
Seed	1	11.821	0.001	0.156
Seed × group	2	2.728	0.073	0.079
Target region	6.215	33.157	<0.001	0.341
Seed × hemisphere	1	2.967	0.090	0.044
Seed × target region	5.845	20.253	<0.001	0.240
Hemisphere × target region	5.078	3.703	0.003	0.055
Hemisphere × target region × age	8	2.067	0.037	0.031
Seed × hemisphere × target region	5.344	2.868	0.013	0.043
Seed × hemisphere × target region × age	8	2.785	0.015	0.042
Age	1	9.433	0.003	0.128
Time	1	0.025	0.876	<0.001
Time × group	2	1.932	0.153	0.057

### Lateralization

**Figure 2:**
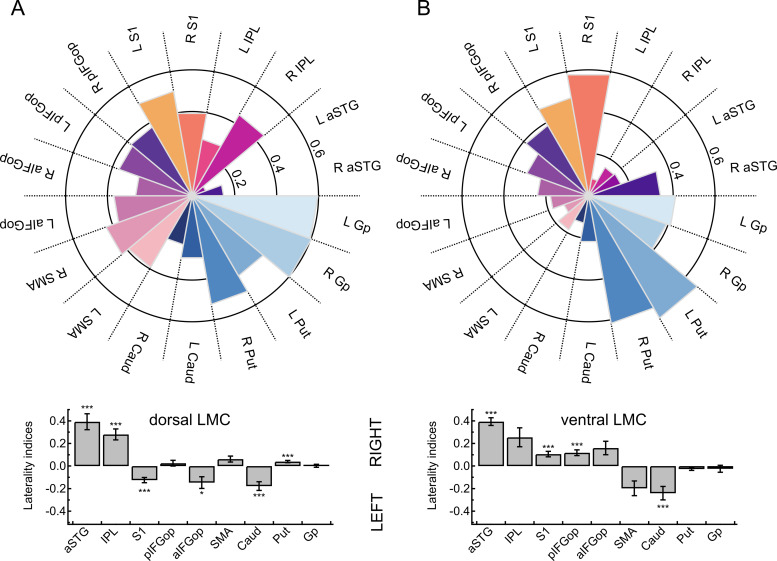
**Connection probability fingerprints and hemispheric lateralization of two laryngeal motor representations.** (**A**) Connectivity fingerprints show the likelihood (0–1) of the dLMC and the (**B**) ventral laryngeal motro cortex (vLMC) averaged per target region across all participants and all sessions. Bar plots indicate hemispheric lateralization at ***P < 0.001 and *P < 0.05 (Bonferroni-corrected). aSTG, anterior superior temporal gyrus; Caud, nucleus caudatus; Gp, globus pallidus; pIFGop, posterior inferior frontal gyrus pars opercularis; aIFGop, anterior inferior frontal gyrus pars opercularis; IPL, inferior parietal lobule; Put, putamen; S1, somatosensory cortex; SMA, supplementary motor area.

Paired *t*-tests assessing the lateralization of dLMC target regions found aSTG (*t *=* *5.59, *P *<* *0.001), IPL (*t *=* *5.80, *P *<* *0.001) and Put (*t *=* *3.94, *P *=* *0.002) to show greater right-hemispheric connectivity and S1 (*t *=* *−5.31, *P *<* *0.001), aIFGop (*t *=* *−2.88, *P *<* *0.048) and Caud (*t *=* *−4.64, *P *<* *0.001) to show greater left-hemispheric connectivity. Paired *t*-tests assessing the lateralization of vLMC target regions found aSTG (*t *=* *8.76, *P *<* *0.001), S1 (*t *=* *4.45, *P *<* *0.001) and pIFGop (*t *=* *4.55, *P *<* *0.001) to show greater right-hemispheric connectivity and Caud (*t *=* *−4.05, *P *<* *0.001) to show greater left-hemispheric connectivity. Figure 2 shows LIs separated for seed and target regions.

Paired *t*-tests assessing whether lateralization differed between dLMC and vLMC were significant for S1 (*t *=* *−6.90, *P *<* *0.001), IFGtr (*t *=* *−3.89, *P *=* *0.001), SMA (*t *=* *3.70, *P *=* *0.003) and Put (*t *=* *4.22, *P *<* *0.001) and marginal significant for pIFGop (*t *=* *−2.72, *P *=* *0.067).

Connection probability fingerprints and hemispheric lateralization of two laryngeal motor representations are shown in Fig. 2.

### Within network analyses

**Figure 3:**
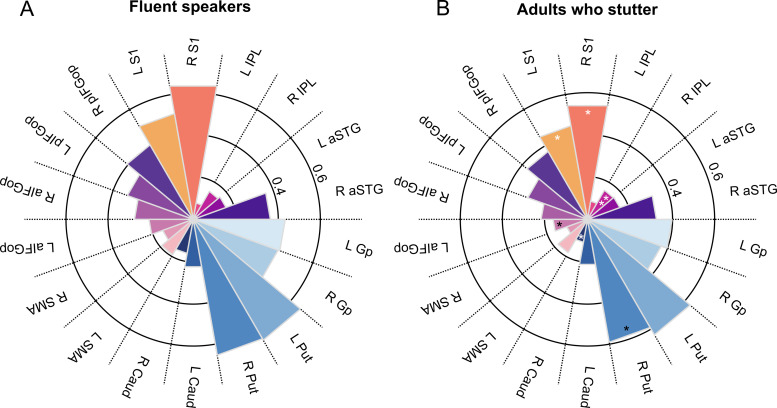
**Connection probability fingerprints of the vLMC.** (**A**) Fingerprints are plotted separately for fluent speakers and (**B**) adults who stutter. Asterisks indicate significant positive (white) and negative (black) relationship between stuttering severity (SSI-4 total score) and structural connectivity with **P < 0.01 and *P < 0.05.

**Figure 4:**
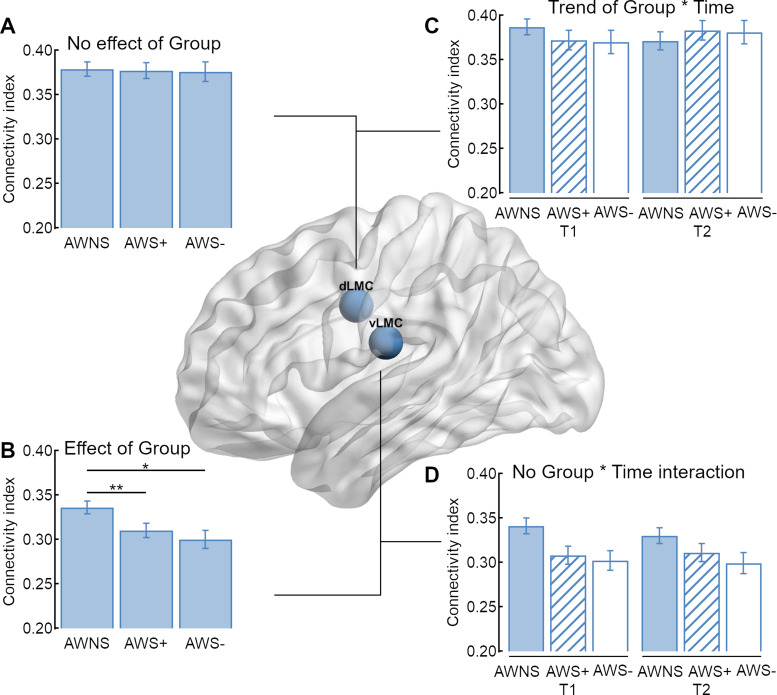
**Structural connectivity of the two laryngeal motor cortices.** (**A**) In sum, adults who stutter (AWS) and AWNS have an overall comparable connectivity index of the dLMC network, (**B**) but AWS have a decreased overall connectivity index of the vLMC network compared to AWNS. (**C**) The trending interaction of Group × Time was not driven by an increase of the overall connectivity index of AWS with intensive stuttering intervention (AWS+), but by a trending decrease in AWNS. (**D**) Time had no influence on the overall structural connectivity of the vLMC network.

Because the global ANCOVA revealed a main effect of Seed, we examined the connection strength of the two seed regions separately. The 3 × 2 × 2 × 9 ANCOVA performed on the connection indices of vLMC revealed a main effect of group with *F*(2,64) = 4.843, *P* = 0.011 such that vLMC connectivity in AWNS, mean (*M*) = 0.336, 95% CI* *=* *[0.321–0.350] was higher than in AWS+, *M* = 0.309, 95% CI* *=* *[0.293–0.326] and AWS−, *M* = 0.301, 95% CI* *= [0.282–0.321] (Figs. 3A,B and 4B). *Post-hoc* group comparisons revealed significant differences between AWNS and AWS+ with a mean difference of 0.035 ± 0.130 SEM, *P* = 0.008 and AWNS and AWS− with a mean difference of 0.027 ± 0.011, *P* = 0.018. Asterisks indicate significantly positive (white) and negative (black) relationship between stuttering severity (SSI-4 score) and structural connectivity with **P < 0.01 and *P < 0.05± 0.013, *P* = 0.545. Figure 3 shows the vLMC connectivity fingerprints for AWS pooled across AWS+, AWS− and AWNS. There was also a main effect of Target region with *F*(8,64) = 41.738, *P* < 0.001, $\eta_{p}^{2}$ =0.395, a main effect of age with *F*(1,64) = 6.500, *P* = 0.013, $\eta_{p}^{2}$ =0.092 and $\eta_{p}^{2}$ =0.131, an interaction of Hemisphere × Target region × Age with *F*(8,64) = 3.672, *P* < 0.001 and $\eta_{p}^{2}$ =0.054, and a trending interaction of Hemisphere × Target region with *F*(4.513,64) = 2.239, *P* = 0.057 and $\eta_{p}^{2}$ =0.034. All other main effects and interactions were not significant (*P* > 0.1, in all cases).

The 3 × 2 × 2 × 9 ANCOVA performed on the connection strength of dLMC revealed a main effect of Target region with *F*(5.676,64) = 18.429, *P* < 0.001 and $\eta_{p}^{2}$ =0.224, an interaction of Hemisphere × Target region with *F*(5.152,64) = 4.568 and *P* < 0.001, $\eta_{p}^{2}$ =0.067, a main effect of age with *F*(1,64) = 6.293, *P* = 0.01 and $\eta_{p}^{2}$ = 0.090 and a trend towards an interaction of Time × Group with *F*(2,64) = 2.654, *P* = 0.078 and $\eta_{p}^{2}$ = 0.077. All other main effects and interactions were not significant (*P* > 0.1, in all cases). To test the interaction of Group × Time, we calculated further *post-hoc* ANCOVAs. For AWNS, the analysis revealed a trending effect of time with *F*(1,24) = 3.257, *P* = 0.082 and $\eta_{p}^{2}$ = 0.119, but no significant effect or trend was found for the other two groups. Figure 4C shows a trend towards a decreased overall structural connectivity in AWNS.

### Regression analyses

We constructed two statistical models incorporating the connection probability of vLMC and dLMC, respectively, in this cohort of 40 adults with chronic persistent stuttering since childhood. We found that connection probability of the vLMC predicted motor signs of stuttering severity, as measured with the SSI-4, over and above the biological factors age, sex and handedness. Variance of SSI-4 total scores in the cohort was explained with *ΔR2 *=* *0.648, *F*[18,36] = 2.625 and *P *=* *0.022; total *R2 *=* *0.754, *F*[21,39] = 2.779 and *P *=* *0.018. In contrast, connection probability of the dLMC did not predict stuttering severity over and above the biological variates *ΔR2 *=* *0.612, *F*[18,36] = 1.834 and *P *=* *0.099; total *R2 *=* *0.681, *F*[21,39] = 1.920 and *P *=* *0.088. Table 4 summarizes the results of the two hierarchical regression analyses.

**Table 4:** Connection probability of vLMC predicts SSI-4stuttering-severity

	*vLMC*		*dLMC*	
Predictor	*ΔR²*	*Standardized β*	*ΔR²*	*Standardized β*
**Step 1**	0.070		0.070	
Age		0.021		0.021
Handedness		0.156		0.156
Sex^a^		0.222		0.222
**Step 2**	0.648^*^		0.612	
Age		0.312		−0.105
Handedness		−0.071		0.574^**^
Sex^a^		−0.205		0.245
Right S1		0.352^*^		0.445^*^
Left S1		0.408^*^		0.432
Right pIFGop		−0.017		−0.131
Left pIFGop		0.274		−0.143
Right aIFGop		0.065		−0.037
Left aIFGop		−0.428^*^		−0.486
Right aSTG		−0.116		0.133
Left aSTG		−0.092		0.188
Right IPL		0.651^**^		0.001
Left IPL		0.085		−0.149
Right SMA		0.352		0.358
Left SMA		0.124		0.071
Right Putamen		−0.594^*^		0.577
Left Putamen		0.160		0.182
Right Caudate		0.587^*^		0.160
Left Caudate		−0.088		−0.369
Right Globus pallidus		0.164		−0.426
Left Globus pallidus		−0.499		0.546
Total *R*²	0.754^*^		0.681	

a Dummy-coded, males = 1, females = −1.

* *P* < 0.05,

** *P* < 0.01.

## Discussion

One essential component of natural fluent speech is the flexible control of pitch and voicing. This speech function is distributed to two laryngeal representations per hemisphere, the dLMC and vLMC. Here, we show that (i) these cortical representations diverge in their structural connectivity profiles, (ii) the dLMC network shares denser connections compared to the vLMC network, (iii) the vLMC connectivity is stronger in fluent speakers compared to adults who stutter, (iv) the connectivity profile of the vLMC predicts stuttering severity and (v) neither of the two structural LMC networks changed with fluency shaping, a common stuttering intervention with a remarkable change of voice control during speaking.

Our findings indicate that the dLMC has an overall stronger structural connectivity compared to the vLMC. This is in line with a neuroimaging study, characterizing the cortical microstructure underlying the two laryngeal representations with quantitative MRI (Eichert *et al*., 2020). With multi-parameter mapping and myelin mapping, (Eichert *et al.,* 2020) found that the dLMC has a myelin content and a cortical thickness that equals that of the primary motor cortex. Furthermore, myelin content and cortical thickness of the dLMC were higher compared to that of the vLMC (Eichert *et al*., 2020). The authors discuss their finding in the context of the evolutionary ‘duplication and migration’ hypothesis (Belyk and Brown, 2017; Jarvis, 2019) and conclude that their findings suggest a primary role of the dLMC for laryngeal motor control in primary motor cortex. Although, diffusion-weighted tractography is widely accepted as a valid method to assess white-matter connectivity *in vivo* in humans (Haber *et al.*, 2020), it is important to keep in mind that various caveats bias tractography data (Van Essen *et al.*, 2014) and validation by invasive studies is desirable. Still, in light of the findings from cortical myelin mapping of the laryngeal representations (Eichert *et al*., 2020), our finding of diverging connectivity profiles of the two laryngeal representations with the dLMC to show a denser structural network compared to the vLMC seems plausible. 

Notably, diffusion tractography is limited in accuracy by inherent uncertainties (Schilling *et al.*, 2019). DTI tractography is capable of providing true neuroanatomical connectivity on the scale of major cortical regions, but it is less reliable at estimating voxel-wise connectivity (Gao *et al.*, 2013; Knösche *et al.*, 2015). Inter-areal connection strength of tractography predicts *ex vivo* tracer connectivity (Donahue *et al.*, 2016) only on a moderate level (Schilling *et al.*, 2019). Differences in accuracy depend in particular on path length, connection strength and the complexity of the pathway. Fibers with crossing, kissing, fanning and curving configurations have been a subject of concern contributing to false-positive (Maier-Hein *et al.*, 2017) and false-negative connections (Aydogan *et al.*, 2018). With this in mind, it is important to acknowledge that the vLMC is located in a cortical region that exhibits a more complex cortical folding compared to the dLMC, which makes probabilistic diffusion tracking more prone to uncertainties.

Differential patterns of structural connectivity of the two laryngeal representations also include hemispheric lateralization. Both LMCs demonstrated right lateralization of the superior temporal gyrus, and left lateralization of the caudate nucleus, which is consistent with the directions reported in the previous report on dLMC connectivity (Kumar *et al.*, 2016). In addition, while the dLMC demonstrated left lateralization of the anterior portion of the inferior frontal gyrus pars opercularis, also consistent with the previous report, the somatosensory cortex, the posterior portion of the inferior frontal gyrus pars opercularis and the somatosensory cortex were right lateralized for vLMC. This heterogeneity also with respect to a hemispheric specialization supports the idea of a functional dissociation of the two laryngeal representations (Belyk and Brown, 2017; Dichter *et al.*, 2018; Eichert *et al*., 2020). In particular, the modulation of pitch in speech and singing has been suggested to be primarily controlled via the dLMC (Dichter *et al.*, 2018; Eichert *et al*., 2020). The causal inference with transcranial magnetic stimulation demonstrated, for example, that in particular the laryngeal representation in the right hemisphere is involved in vocal pitch regulation (Finkel *et al.*, 2019) and auditory pitch discrimination (Sammler *et al.*, 2015). However, currently the ground truth of the anatomical connectivity of LMC to laryngeal motor neurons and cortical and sub-cortical brain areas results from tracing studies of a single cortical motor representation in mammals and humans (Kuypers, 1958; Kirzinger and Jürgens, 1982; Simonyan and Jürgens, 2002; Simonyan, 2014). Thus, neurophysiological and brain stimulation studies (Hamdy *et al.*, 1998) might be advantageous to map out the particular connectivity of a dual representation in humans, and to foster the distinct roles of dLMC and vLMC in concordant and specific larynx functions.

A functional dissociation of the laryngeal representations is further suggested by the varying involvement of these two areas in persistent developmental stuttering. This speech fluency disorder is characterized by a white-matter deficit, i.e. a reduced fractional anisotropy, in the left vLMC (Sommer *et al.*, 2002; Chang *et al.*, 2008; Watkins *et al.*, 2008) and most likely involves fibers of the superior longitudinal fasciculus/arcuate fasciculus (Connally *et al.*, 2014; Neef *et al.*, 2015, 2018; Kronfeld-Duenias *et al.*, 2016, 2018). The white-matter deficit might cause a disconnection of the ventral laryngeal motor representation from left perisylvian speech regions (Sommer *et al.*, 2002). Here, we substantiated this longstanding finding by showing that adults who stutter have a reduced overall connection probability of the vLMCs when compared to fluent speakers. Moreover, structural connectivity profiling of both laryngeal motor representations revealed that only vLMC structural connectivity serves as a powerful statistical predictor of stuttering severity. In particular, connection probability of the left vLMC with the left primary somatosensory cortex and inferior gyrus pars opercularis, and connectivity of the right vLMC with the right primary somatosensory cortex, inferior parietal lobe, putamen and caudate nucleus strongly related to the motor signs of stuttering. The involvement of frontal and parietal sites substantiates the assumption that stuttering results from an insufficient feed forward and feedback control during speech-related sensorimotor signal transmission (Guenther, 2016). In addition, our findings further affirm that affected circuits extend beyond the known left hemisphere speech motor pathways (Kronfeld-Duenias *et al.*, 2018; Neef *et al.*, 2018) and engage the basal ganglia system (Alm, 2004; Connally *et al.*, 2018; Chang and Guenther, 2019).

Both LMC networks established strong connections with cortical brain areas specified to process planning and timing of motor sequences, sensory input and feedback and sensorimotor integration. Strikingly, only the ventral laryngeal representation is affected in stuttering. Our vLMC seed coordinate was derived from the first dMRI study on stuttering (*x* = −48, *y* = −15 and *z* = 18) (Sommer *et al.*, 2002). This white-matter site is closely located to sites of cortical activity reported for tasks that were designed to stimulate and differentiate dLMC and vLMC brain activity during whistling and singing (*x* = −59, *y* = −16 and *z* = 13) (Belyk *et al*., 2020) or vocalization and vowel production (*x* = −58, *y* = −2 and *z* = 20) (Eichert *et al*., 2020). Similarly, earlier fMRI studies that investigated vowel production (Grabski *et al.*, 2012), vocal imitation (Belyk *et al.*, 2016), pitch (Peck *et al.*, 2009), cough (Mazzone *et al.*, 2011) and brain alterations in spasmodic dysphonia (Simonyan and Ludlow, 2012) relate laryngeal control to our chosen vLMC coordinate. Cyto- and myeloarchitecture of the ventral laryngeal representation is currently unknown and it has been suggested that this region might belong to the cytoarchitectonic area 6 (Pfenning *et al.*, 2014; Dichter *et al.*, 2018; Jarvis, 2019; Eichert *et al*., 2020) or to the cytoarchitectonic area 43 (Belyk and Brown, 2017; Belyk *et al*., 2020). Besides this dissent, different research groups seem to agree on the idea that the vLMC does not belong to the primary motor cortex. The assignment of the two laryngeal representations to different architectural areas underpins the suggestion of segregated functions. However, it remains unclear how the vLMC contributes distinctively to larynx control.

One striking phenomenon in stuttering is the preserved ability to sing. In contrast, speech prosody and pitch control are apparently disrupted during stuttering (Neumann *et al.*, 2018). Both vocal functions, singing and speaking, involve a dedicated control of laryngeal muscles to regulate pitch and voicing and to coordinate vocalization with articulation and breathing. Similarly, both functions rely on shared cognitive processes and large-scale networks that overlap to a great extent (Özdemir *et al.*, 2006). However, a theoretical discussion infers that musical pitch requires a more accurate encoding to ensure discrete melody production than does speech for which pitch variation is continuous (Zatorre and Baum, 2012). Accordingly, the reduced degrees of freedom for pitch modulation in singing provide a finer and more specified template of upcoming vocalizations. Such a temporal specification that also includes rhythm in song might facilitate fluency as observed when affected individuals sing. The same reasoning holds true for other fluency-enhancing techniques such as chorus reading and metronome speaking (Barber, 1940; Wingate, 1969; Davidow *et al.*, 2009), carry-over fluency induced by extreme prolongations (Briley *et al.*, 2016) or reduced voicing complexity by fluency-shaping (Euler *et al.*, 2009). It seems plausible to assume that network formation for speech and song production is dynamic and task-dependent and varies in concert with involved brain regions that perform parallel computations. Singing might recruit network formations with a widely intact structural organization biased by a stronger dLMC involvement, while speaking might more heavily involve vLMC networks, which are affected in stuttering. A different interpretation can be drawn from a recent case study. Two expert musicians underwent awake craniotomy surgery. The stimulation of the vLMC area disrupted speech and music production, i.e. playing the piano or the guitar (Leonard *et al.*, 2019). The authors suggest that this ventral area might code more complex representations that are independent of specific effectors such as laryngeal muscles.

This analysis revealed no impact of an intensive fluency-shaping intervention on white-matter networks of the two laryngeal representations. This result is somewhat counterintuitive because the speech-restructuring method required individuals who stutter to learn a changed speech pattern. This speech pattern comprised soft voice onsets, consonant lenitions and controlled sound prolongations. Thus, voicing and timing were the key features under change over the course of the acquisition of the new speech technique (Euler *et al.*, 2009). In contrast to unchanged white-matter structures of voice control, resting-state connectivity was strengthened within the dLMC network (Korzeczek et al., 2020). Specifically, intervention synchronized resting-state activity between the left dLMC and the left posterior portion of the inferior frontal gyrus pars opercularis, the left inferior parietal lobe and the right posterior superior temporal gyrus. This observation is in line with a previous study that investigated speaking-related fMRI network connectivity of the very same therapy program, and similarly observed an increased auditory-motor coupling between the aSTG and the ventral articulatory motor cortex (Kell *et al.*, 2018). However, the observation that the structural dLMC network is unaffected in stuttering, but recruited by stuttering intervention suggests a compensatory involvement of these networks in assisted recovery. This is a new finding that contradicts the previous reports on an intervention-induced normalization of brain activity (Neumann *et al.*, 2005, 2018; Kell *et al.*, 2009, 2018). It seems that functional plasticity does not translate into changed structural connectivity, results in therapy-related changes in both resting-state and functional connectivity during speaking involving other regions than the vLMC. This indeed does not reflect a normalization of functional connectivity, but goes along with normalized activation patterns during speaking (Kell *et al.*, 2009).

## Conclusion

In sum, our findings strongly support the view of a functional segregation of the dual cortical larynx representations, which is based on a diverging structural network organization. The dorsal laryngeal representation has an overall denser structural network compared to the ventral one. The intra-hemispheric connectivity profiles of bilateral ventral laryngeal representations predict motor signs of stuttering over and above the biological variates age, sex and handedness, and serve as a weighty neuronal trait marker of stuttering. However, the vLMC network is insensitive to intensive fluency-shaping, i.e. shows no structural neuroplasticity after restructured pitch and voicing in speech.

## Abbreviations

aIFGop =: anterior inferior frontal gyrus pars opercularis
aSTG =: anterior superior temporal gyrus
AWNS =: adults who do not stutter
AWS− =: adults who stutter and did not participate in the intervention
AWS+ =: adults who stutter and participated in the intervention
Caud =: caudate nucleus
dLMC =: dorsal laryngeal motor cortex
Gp =: globus pallidus
IPL =: inferior parietal lobe
OASES =: Overall Assessment of the Speaker’s Experience of Stuttering
OP =: parietal operculum
Pfm =: inferior parietal lobule area
Pga =: inferior parietal lobule area
pIFGop =: posterior inferior frontal gyrus pars opercularis
Put =: putamen
S1 =: primary somatosensory cortex
SMA =: supplementary motor area
SSI-4 =: Stuttering Severity Index
T1 =: test time one
T2 =: test time two
vLMC =: ventral laryngeal motor cortex
